# Supplementary material for: The Dynamics of Antimicrobial Resistance among Enterobacteriaceae Isolates in Russia: Results of the 2012–2018 INFORM and ATLAS International Program Studies
Source: Antibiotics (Basel). 2022 Jun 10;11(6):790. doi: 10.3390/antibiotics11060790 (PMC9220778; doi:10.3390/antibiotics11060790)
Supplement: Supplementary file 1 [file antibiotics-11-00790-s001.zip › antibiotics-1710558-supplementary.pdf]

Supplementary Materials

Table S1. Distribution of Enterobacterales based upon source of infection (an alternative variant of Table 3).

**Species**	**Total**	**Intra-Abdominal Infections**	**Genitourinary Tract Infections**	**Skin and soft tissue Infections**	**Lower Respiratory Tract Infections**
**All isolates**	**N=3,811**	**N=614**	**N=1,069**	**N=894**	**N=1,234**
Klebsiella spp.	1464 [100%]	186 [12.7%]	287 [19.6%]	274 [18.7%]	717 [49%]
Escherichia coli	1314 [100%]	265 [20.2%]	502 [38.2%]	329 [25%]	218 [16.6%]
Enterobacter spp.	335 [100%]	60 [17.9%]	74 [22.1%]	102 [30.4%]	99 [29.6%]
Proteus spp.	324 [100%]	33 [10.2%]	116 [35.8%]	94 [29%]	81 [25%]
Citrobacter spp.	142 [100%]	34 [23.9%]	33 [23.2%]	43 [30.3%]	32 [22.5%]
Serratia spp.	108 [100%]	11 [10.2%]	17 [15.7%]	21 [19.4%]	59 [54.6%]
Morganella spp.	85 [100%]	16 [18.8%]	31 [36.5%]	19 [22.4%]	19 [22.4%]
Providencia spp.	25 [100%]	7 [28%]	8 [32%]	9 [36%]	1 [4%]
Raoultella spp.	10 [100%]	1 [10%]	1 [10%]	2 [20%]	6 [60%]
Hafnia spp.	3 [100%]	1 [33.3%]	0 [0%]	0 [0%]	2 [66.7%]
Pluralibacter spp.	1 [100%]	0 [0%]	0 [0%]	1 [100%]	0 [0%]

Table S2. MIC50 (mg/L) values for *Enterobacterales*.

Antimicrobial	Over all time	2012	2013	2014	2015	2016	2017	2018
**Amikacin**	2	4	4	4	2	2	2	2
**Gentamicin**	0.5	-	-	-	-	-	-	0.5
**Doripenem**	0.06	0.12	0.06	0.06	0.06	0.06	0.03	-
**Ertapenem**	0.06	0.06	0.06	-	-	-	-	-
**Imipenem**	0.25	0.25	0.25	0.25	0.25	0.25	0.25	0.5
**Meropenem**	0.06	0.06	0.03	0.06	0.06	0.06	0.03	0.06
**Cefepime**	1	8	4	4	0.12	0.12	0.5	2
**Cefoperazone/sulbactam**	4	-	-	-	-	-	-	4
**Ceftaroline**	16	32	128	256	0.25	0.5	16	16
**Ceftaroline/avibactam**	0.06	0.12	0.06	0.06	-	-	-	-
**Ceftazidime**	1	8	2	2	0.25	0.5	0.5	4
**Ceftazidime/avibactam**	0.12	0.25	0.12	0.12	0.12	0.12	0.12	0.25
**Ceftolozane/tazobactam**	0.25	-	-	-	-	-	0.25	-
**Ceftriaxone**	0.5	-	-	-	-	0.25	12	-
**Trimethoprim sulfa**	2	-	-	-	-	-	-	2
**Tigecycline**	0.5	0.5	0.5	0.5	0.25	0.25	0.25	0.5
**Colistin**	0.5	-	-	1	0.5	0.25	0.5	0.5
**Aztreonam**	1	8	4	8	0.12	0.25	1	6
**Aztreonam avibactam**	0.06	0.06	0.06	0.06	0.06	0.06	0.03	0.06
**Ampicillin/sulbactam**	32	-	-	-	-	-	-	32
**Amoxicillin/clavulanate**	16	16	16	16	16	16	16	32
**Ampicillin**	64	64	64	64	64	64	64	32
**Piperacillin/tazobactam**	4	4	4	4	2	4	2	4
**Ciprofloxacin**	1	-	-	-	-	-	-	1
**Levofloxacin**	0.5	2	0.5	1	0.06	0.25	0.25	1

Table S3. MIC90 (mg/L) dynamics for Enterobacterales

Antimicrobial	Over all time	2012	2013	2014	2015	2016	2017	2018
**Amikacin**	64	64	16	16	16	8	16	128
**Gentamicin**	32	-	-	-	-	-	-	32
**Doripenem**	0.5	1	0.25	0.25	0.25	0.25	0.5	-
**Ertapenem**	1	1	1	-	-	-	-	-
**Imipenem**	4	4	2	2	4	2	2	16
**Meropenem**	0.25	0.25	0.12	0.25	0.12	0.12	0.25	32
**Cefepime**	32	32	32	32	32	32	32	64
**Cefoperazone/sulbactam**	128	-	-	-	-	-	-	128
**Ceftaroline**	256	256	256	256	256	256	256	16
**Ceftaroline/avibactam**	0.25	0.5	0.25	0.25	-	-	-	-
**Ceftazidime**	256	256	128	128	128	128	256	256
**Ceftazidime/avibactam**	1	1	0.5	0.5	0.5	0.5	0.5	2
**Ceftolozane/tazobactam**	64	-	-	-	-	-	64	-
**Ceftriaxone**	32	-	-	-	-	32	32	-
**Co-trimoxazole**	64	-	-	-	-	-	-	64
**Tigecycline**	2	4	2	2	1	1	1	2
**Colistin**	8	-	-	8	16	16	16	16
**Aztreonam**	256	256	256	128	128	128	256	128
**Aztreonam/avibactam**	0.25	0.5	0.25	0.12	0.12	0.12	0.12	0.25
**Ampicillin/sulbactam**	128	-	-	-	-	-	-	128
**Amoxicillin/clavulanate**	32	32	32	32	64	64	64	32
**Ampicillin**	64	64	64	64	64	64	64	32

**Piperacillin/tazobactam**	256	256	256	128	256	256	256	128
**Ciprofloxacin**	8	-	-	-	-	-	-	8
**Levofloxacin**	16	8	8	8	16	16	16	16

Table S4. The MDR rates depending on source of infection.

According to the criteria proposed by Hackel 2016					
**Year**	**Total**	**IAIs**	**UTIs**	**SSTIs**	**LRTIs**
**All Enterobacterales**					
2012	203 / 394 (51.5%)	8 / 17 (47.1%)	62 / 167 (37.1%)	52 / 98 (53.1%)	81 / 112 (72.3%)
2013	310 / 712 (43.5%)	33 / 84 (39.3%)	81 / 198 (40.9%)	79 / 198 (39.9%)	117 / 232 (50.4%)
2014	343 / 812 (42.2%)	43 / 154 (27.9%)	105 / 262 (40.1%)	55 / 137 (40.1%)	140 / 259 (54.1%)
2015	112 / 383 (29.2%)	3 / 17 (17.6%)	33 / 133 (24.8%)	30 / 105 (28.6%)	46 / 128 (35.9%)
2016	160 / 478 (33.5%)	13 / 89 (14.6%)	30 / 100 (30%)	55 / 131 (42%)	62 / 158 (39.2%)
2017	186 / 470 (39.6%)	25 / 91 (27.5%)	23 / 83 (27.7%)	52 / 123 (42.3%)	86 / 173 (49.7%)
2018	269 / 562 (47.9%)	43 / 162 (26.5%)	62 / 126 (49.2%)	49 / 102 (48%)	115 / 172 (66.9%)
Total	1583 / 3811 (41.5%)	168 / 614 (27.4%)	396 / 1069 (37%)	372 / 894 (41.6%)	647 / 1234 (52.4%)
E. coli					
2012	44 / 123 (35.8%)	3 / 7 (42.9%)	19 / 72 (26.4%)	10 / 23 (43.5%)	12 / 21 (57.1%)
2013	80 / 253 (31.6%)	17 / 47 (36.2%)	25 / 102 (24.5%)	27 / 69 (39.1%)	11 / 35 (31.4%)
2014	75 / 304 (24.7%)	17 / 79 (21.5%)	36 / 136 (26.5%)	15 / 50 (30%)	7 / 39 (17.9%)
2015	31 / 145 (21.4%)	2 / 8 (25%)	16 / 68 (23.5%)	8 / 43 (18.6%)	5 / 26 (19.2%)
2016	40 / 165 (24.2%)	2 / 29 (6.9%)	10 / 45 (22.2%)	20 / 55 (36.4%)	8 / 36 (22.2%)
2017	43 / 161 (26.7%)	7 / 33 (21.2%)	6 / 33 (18.2%)	18 / 55 (32.7%)	12 / 40 (30%)
2018	57 / 163 (35%)	24 / 62 (38.7%)	13 / 46 (28.3%)	11 / 34 (32.4%)	9 / 21 (42.9%)
Total	370 / 1314 (28.2%)	72 / 265 (27.2%)	125 / 502 (24.9%)	109 / 329 (33.1%)	64 / 218 (29.4%)
K. pneumoniae					
2012	100 / 129 (77.5%)	2 / 3 (66.7%)	19 / 33 (57.6%)	25 / 33 (75.8%)	54 / 60 (90%)
2013	152 / 232 (65.5%)	12 / 24 (50%)	30 / 42 (71.4%)	31 / 47 (66%)	79 / 119 (66.4%)
2014	199 / 309 (64.4%)	19 / 37 (51.4%)	46 / 75 (61.3%)	32 / 52 (61.5%)	102 / 145 (70.3%)
2015	51 / 108 (47.2%)	1 / 3 (33.3%)	12 / 32 (37.5%)	14 / 23 (60.9%)	24 / 50 (48%)
2016	80 / 135 (59.3%)	9 / 27 (33.3%)	14 / 21 (66.7%)	15 / 20 (75%)	42 / 67 (62.7%)
2017	104 / 145 (71.7%)	13 / 21 (61.9%)	9 / 13 (69.2%)	24 / 30 (80%)	58 / 81 (71.6%)
2018	148 / 188 (78.7%)	12 / 29 (41.4%)	24 / 29 (82.8%)	23 / 24 (95.8%)	89 / 106 (84%)
Total	834 / 1246 (66.9%)	68 / 144 (47.2%)	154 / 245 (62.9%)	164 / 229 (71.6%)	448 / 628 (71.3%)
According to the criteria proposed by Castanheira 2019					
**Year**	**Total**	**IAI**	**UTI**	**SSTI**	**LRTI**
***All* Enterobacterales**					
2012	59 / 394 (15%)	1 / 17 (5.9%)	18 / 167 (10.8%)	18 / 98 (18.4%)	22 / 112 (19.6%)
2013	19 / 712 (2.7%)	2 / 84 (2.4%)	7 / 198 (3.5%)	4 / 198 (2%)	6 / 232 (2.6%)
2014	66 / 812 (8.1%)	3 / 154 (1.9%)	26 / 262 (9.9%)	11 / 137 (8%)	26 / 259 (10%)
2015	23 / 383 (6%)	0 / 17 (0%)	3 / 133 (2.3%)	9 / 105 (8.6%)	11 / 128 (8.6%)
2016	28 / 478 (5.9%)	8 / 89 (9%)	3 / 100 (3%)	10 / 131 (7.6%)	7 / 158 (4.4%)
2017	33 / 470 (7%)	3 / 91 (3.3%)	10 / 83 (12%)	9 / 123 (7.3%)	11 / 173 (6.4%)
2018	111 / 562 (19.8%)	8 / 162 (4.9%)	31 / 126 (24.6%)	18 / 102 (17.6%)	54 / 172 (31.4%)
Total	339 / 3811 (8.9%)	25 / 614 (4.1%)	98 / 1069 (9.2%)	79 / 894 (8.8%)	137 / 1234 (11.1%)
E. coli					
2012	10 / 123 (8.1%)	1 / 7 (14.3%)	3 / 72 (4.2%)	3 / 23 (13%)	1 / 21 (4.8%)
2013	3 / 253 (1.2%)	0 / 47 (0%)	1 / 102 (1%)	2 / 69 (2.9%)	0 / 35 (0%)
2014	0 / 304 (0%)	0 / 79 (0%)	0 / 136 (0%)	0 / 50 (0%)	0 / 39 (0%)
2015	2 / 145 (1.4%)	0 / 8 (0%)	0 / 68 (0%)	2 / 43 (4.7%)	0 / 26 (0%)
2016	1 / 165 (0.6%)	0 / 29 (0%)	0 / 45 (0%)	0 / 55 (0%)	0 / 36 (0%)
2017	0 / 161 (0%)	0 / 33 (0%)	0 / 33 (0%)	1 / 55 (1.8%)	0 / 40 (0%)
2018	7 / 163 (4.3%)	3 / 62 (4.8%)	3 / 46 (6.5%)	0 / 34 (0%)	3 / 21 (14.3%)
Total	23 / 1314 (1.8%)	4 / 265 (1.5%)	7 / 502 (1.4%)	8 / 329 (2.4%)	4 / 218 (1.8%)
K. pneumoniae					
2012	22 / 129 (17.1%)	0 / 3 (0%)	1 / 33 (3%)	9 / 33 (27.3%)	12 / 60 (20%)
2013	7 / 232 (3%)	1 / 24 (4.2%)	1 / 42 (2.4%)	1 / 47 (2.1%)	4 / 119 (3.4%)
2014	25 / 309 (8.1%)	1 / 37 (2.7%)	9 / 75 (12%)	7 / 52 (13.5%)	8 / 145 (5.5%)
2015	10 / 108 (9.3%)	0 / 3 (0%)	1 / 32 (3.1%)	4 / 23 (17.4%)	5 / 50 (10%)
2016	17 / 135 (12.6%)	5 / 27 (18.5%)	2 / 21 (9.5%)	5 / 20 (25%)	5 / 67 (7.5%)
2017	15 / 145 (10.3%)	2 / 21 (9.5%)	3 / 13 (23.1%)	4 / 30 (13.3%)	6 / 81 (7.4%)

2018	80 / 188 (42.6%)	3 / 29 (10.3%)	17 / 29 (58.6%)	12 / 24 (50%)	48 / 106 (45.3%)
Total	176 / 1246 (14.1%)	12 / 144 (8.3%)	34 / 245 (13.9%)	42 / 229 (18.3%)	88 / 628 (14%)

Table S5. The cumulative number of ESBL-positive (ESBL+) strains among the isolates depending on bacterial species and location of the infection source.

Species	N(%) ESBL+ isolates (% from total)	N(%) ESBL+ isolates (among IAI)	N(%) ESBL+ isolates (among UTI)	N(%) ESBL+ isolates (among SSTI)	N(%) ESBL+ isolates (among LRTI)
All isolates	1784 / 3811 (46.8%)	220 / 614 (35.8%)	420 / 1069 (39.3%)	406 / 894 (45.4%)	738 / 1234 (59.8%)
Escherichia coli	529 / 1314 (40.3%)	107 / 265 (40.4%)	167 / 502 (33.3%)	147 / 329 (44.7%)	108 / 218 (49.5%)
Klebsiella pneumoniae	935 / 1246 (75%)	85 / 144 (59%)	163 / 245 (66.5%)	177 / 229 (77.3%)	510 / 628 (81.2%)
Enterobacter cloacae	79 / 297 (26.6%)	8 / 48 (16.7%)	20 / 66 (30.3%)	22 / 98 (22.4%)	29 / 85 (34.1%)
Proteus mirabilis	110 / 255 (43.1%)	5 / 24 (20.8%)	37 / 93 (39.8%)	24 / 66 (36.4%)	44 / 72 (61.1%)
Klebsiella oxytoca	20 / 130 (15.4%)	5 / 23 (21.7%)	2 / 25 (8%)	2 / 27 (7.4%)	11 / 55 (20%)
Citrobacter freundii	23 / 100 (23%)	2 / 25 (8%)	6 / 25 (24%)	7 / 27 (25.9%)	8 / 23 (34.8%)
Serratia marcescens	29 / 100 (29%)	3 / 11 (27.3%)	8 / 15 (53.3%)	6 / 21 (28.6%)	12 / 53 (22.6%)
Morganella morganii	15 / 85 (17.6%)	2 / 16 (12.5%)	6 / 31 (19.4%)	5 / 19 (26.3%)	2 / 19 (10.5%)
Klebsiella aerogenes	23 / 76 (30.3%)	/ 13 (0%)	6 / 17 (35.3%)	6 / 17 (35.3%)	11 / 29 (37.9%)
Proteus vulgaris	2 / 56 (3.6%)	0 / 5 (0%)	1 / 20 (5%)	1 / 22 (4.5%)	0 / 9 (0%)
Citrobacter braakii	8 / 24 (33.3%)	1 / 3 (33.3%)	1 / 2 (50%)	5 / 13 (38.5%)	1 / 0 (0%)
Enterobacter asburiae	1 / 16 (6.3%)	0 / 5 (0%)	1 / 3 (33.3%)	0 / 2 (0%)	0 / 6 (0%)
Providencia rettgeri	2 / 15 (13.3%)	0 / 3 (0%)	0 / 2 (0%)	2 / 9 (22.2%)	0 / 1 (0%)
Klebsiella variicola	0 / 12 (0%)	0 / 6 (0%)	0 / 0 (0%)	0 / 1 (0%)	0 / 5 (0%)
Enterobacter, non-speciated	0 / 11 (0%)	0 / 0 (0%)	0 / 5 (0%)	0 / 0 (0%)	0 / 6 (0%)
Proteus hauseri	0 / 10 (0%)	0 / 2 (0%)	0 / 3 (0%)	0 / 5 (0%)	0 / 0 (0%)
Citrobacter koseri	0 / 9 (0%)	0 / 0 (0%)	0 / 3 (0%)	0 / 3 (0%)	0 / 3 (0%)
Raoultella ornithinolytica	0 / 9 (0%)	0 / 1 (0%)	0 / 1 (0%)	0 / 2 (0%)	0 / 6 (0%)
Enterobacter kobei	3 / 6 (50%)	0 / 3 (0%)	0 / 0 (0%)	2 / 2 (100%)	1 / 1 (100%)
Serratia liquefaciens	0 / 6 (0%)	0 / 0 (0%)	0 / 1 (0%)	0 / 0 (0%)	0 / 5 (0%)
Citrobacter farmeri	0 / 5 (0%)	0 / 5 (0%)	0 / 0 (0%)	0 / 0 (0%)	0 / 0 (0%)
Enterobacter ludwigii	0 / 5 (0%)	0 / 4 (0%)	0 / 0 (0%)	0 / 0 (0%)	0 / 1 (0%)
Providencia alcalifaciens	0 / 5 (0%)	0 / 3 (0%)	0 / 2 (0%)	0 / 0 (0%)	0 / 0 (0%)
Providencia stuartii	2 / 5 (40%)	1 / 1 (100%)	1 / 4 (25%)	0 / 0 (0%)	0 / 0 (0%)
Citrobacter amalonaticus	0 / 4 (0%)	0 / 1 (0%)	0 / 3 (0%)	0 / 0 (0%)	0 / 6 (0%)
Hafnia alvei	2 / 3 (66.7%)	1 / 1 (100%)	0 / 0 (0%)	0 / 0 (0%)	1 / 2 (50%)
Proteus penneri	0 / 3 (0%)	0 / 2 (0%)	0 / 0 (0%)	0 / 1 (0%)	0 / 0 (0%)
Pluralibacter gergoviae	0 / 1 (0%)	0 / 0 (0%)	0 / 0 (0%)	0 / 1 (0%)	0 / 0 (0%)
Raoultella planticola	0 / 1 (0%)	0 / 0 (0%)	0 / 0 (0%)	0 / 0 (0%)	0 / 0 (0%)
Serratia odorifera	0 / 1 (0%)	0 / 0 (0%)	0 / 0 (0%)	0 / 0 (0%)	0 / 1 (0%)
Serratia ureilytica	1 / 1 (100%)	0 / 0 (0%)	1 / 1 (100%)	0 / 0 (0%)	0 / 0 (0%)

Table S6. Dynamics of the rate of ESBL-positive isolates depending on source of infection throughout the study period.

Year	Total	IAI	UTI	SSTI	LRTI
***All* Enterobacterales**					
2012	200 / 394 (50.8%)	11 / 17 (64.7%)	56 / 167 (33.5%)	54 / 98 (55.1%)	79 / 112 (70.5%)
2013	372 / 712 (52.2%)	42 / 84 (50%)	90 / 198 (45.5%)	87 / 198 (43.9%)	153 / 232 (65.9%)
2014	443 / 812 (54.6%)	66 / 154 (42.9%)	118 / 262 (45%)	78 / 137 (56.9%)	181 / 259 (69.9%)
2015	134 / 383 (35%)	4 / 17 (23.5%)	36 / 133 (27.1%)	35 / 105 (33.3%)	59 / 128 (46.1%)
2016	198 / 478 (41.4%)	20 / 89 (22.5%)	44 / 100 (44%)	61 / 131 (46.6%)	73 / 158 (46.2%)
2017	197 / 470 (41.9%)	28 / 91 (30.8%)	27 / 83 (32.5%)	56 / 123 (45.5%)	86 / 173 (49.7%)
2018	240 / 562 (42.7%)	49 / 162 (30.2%)	49 / 126 (38.9%)	35 / 102 (34.3%)	107 / 172 (62.2%)
Total	1784 / 3811 (46.8%)	220 / 614 (35.8%)	420 / 1069 (39.3%)	406 / 894 (45.4%)	738 / 1234 (59.8%)
E. coli					
2012	46 / 123 (37.4%)	3 / 7 (42.9%)	17 / 72 (23.6%)	14 / 23 (60.9%)	12 / 21 (57.1%)
2013	109 / 253 (43.1%)	20 / 47 (42.6%)	35 / 102 (34.3%)	35 / 69 (50.7%)	19 / 35 (54.3%)
2014	122 / 304 (40.1%)	32 / 79 (40.5%)	45 / 136 (33.1%)	23 / 50 (46%)	22 / 39 (56.4%)
2015	45 / 145 (31%)	3 / 8 (37.5%)	18 / 68 (26.5%)	14 / 43 (32.6%)	10 / 26 (38.5%)
2016	61 / 165 (37%)	4 / 29 (13.8%)	20 / 45 (44.4%)	23 / 55 (41.8%)	14 / 36 (38.9%)
2017	68 / 161 (42.2%)	12 / 33 (36.4%)	13 / 33 (39.4%)	25 / 55 (45.5%)	18 / 40 (45%)
2018	78 / 163 (47.9%)	33 / 62 (53.2%)	19 / 46 (41.3%)	13 / 34 (38.2%)	13 / 21 (61.9%)
Total	529 / 1314 (40.3%)	107 / 265 (40.4%)	167 / 502 (33.3%)	147 / 329 (44.7%)	108 / 218 (49.5%)
K. pneumoniae					
2012	97 / 129 (75.2%)	3 / 3 (100%)	20 / 33 (60.6%)	23 / 33 (69.7%)	51 / 60 (85%)
2013	191 / 232 (82.3%)	17 / 24 (70.8%)	31 / 42 (73.8%)	35 / 47 (74.5%)	108 / 119 (90.8%)
2014	242 / 309 (78.3%)	24 / 37 (64.9%)	50 / 75 (66.7%)	44 / 52 (84.6%)	124 / 145 (85.5%)
2015	57 / 108 (52.8%)	1 / 3 (33.3%)	13 / 32 (40.6%)	13 / 23 (56.5%)	30 / 50 (60%)
2016	88 / 135 (65.2%)	12 / 27 (44.4%)	16 / 21 (76.2%)	16 / 20 (80%)	44 / 67 (65.7%)
2017	111 / 145 (76.6%)	15 / 21 (71.4%)	9 / 13 (69.2%)	26 / 30 (86.7%)	61 / 81 (75.3%)
2018	149 / 188 (79.3%)	13 / 29 (44.8%)	24 / 29 (82.8%)	20 / 24 (83.3%)	92 / 106 (86.8%)
Total	935 / 1246 (75%)	85 / 144 (59%)	163 / 245 (66.5%)	177 / 229 (77.3%)	510 / 628 (81.2%)
